# The oncogenic and prognostic role of PDK1 in the progression and metastasis of ovarian cancer

**DOI:** 10.7150/jca.47278

**Published:** 2021-01-01

**Authors:** Shasha Yao, Wenwen Shang, Lei Huang, Rui Xu, Ming Wu, Fang Wang

**Affiliations:** 1 Department of Laboratory Medicine, the First Affiliated Hospital of Nanjing Medical University, 210029, Nanjing, China.; 2 National Key Clinical Department of Laboratory Medicine, 210029, Nanjing, China.; 3 Department of Laboratory Medicine, the Affiliated Jiangning Hospital of Nanjing Medical University, 211100, Nanjing, China.

**Keywords:** Pyruvate dehydrogenase kinase 1, ovarian cancer, tumor metabolic, biological behavior, prognostic biomarker

## Abstract

Ovarian cancer (OC) is the most lethal of gynecological tumors in women. Tumor metabolism has become a new opportunity in the treatment of tumors. Pyruvate dehydrogenase kinase 1 (PDK1), as a key regulatory enzyme implicated in metabolic reprogramming of tumors, abnormally high expressed in various tumors and involved in the regulation of tumor cell biological behavior. However, the role of PDK1 in the occurrence and development of ovarian cancer remains unclear. Our team identified the expression of PDK1 in ovarian cancer cell lines and tissues through RT-PCR and immunohistochemical staining and evaluated the correlation of PDK1 expression with clinicopathologic features of patients and survival analyses. We used a variety of *in vitro* experiments to explore the influence of PDK1 on proliferation, invasion, migration, colony formation, apoptosis and the cell cycle of ovarian cancer cell lines CAOV3 and SKOV3. PDK1 was highly expressed in ovarian cancer cell lines and OC tissues. High expression of PDK1 was closely correlated to tumor size, FIGO stage, extraovarian metastases status and distribution. Univariate and multivariate Cox regression analysis identified that PDK1 was an independent prognostic factor for overall survival. Moreover, PDK1 was a superior predictor in prognosis of ovarian cancer and the incorporation of CA125 into PDK1 generated a predictive combination that displayed better predictive accuracy for overall survival. Downregulation of PDK1 suppressed the biological behavior of ovarian cancer cells due to S phase arrest and cellular apoptosis. PDK1 may serve as a novel prognostic biomarker, even a promising antineoplastic target of ovarian cancer.

## Introduction

Ovarian cancer (OC) is the second most common and the most lethal gynecological malignancy with 22,240 new cases and 14,070 deaths estimated in the United States for 2018 1. In China, more than 52,100 patients were newly diagnosed with ovarian cancer, and 22,500 died of ovarian cancer in 2015 2. The diagnosis and clinical treatment such as chemotherapy resistance of advanced ovarian cancer remains a great challenge of medical oncology as few symptoms early occurs in its course, causing the majority of patients diagnosed with advanced stage disease 3, 4. Moreover, poor prognosis for OC patients is still reflected in the < 40% 5-year survival rate and 1.3% lifetime risk 5. Hence, more sensitive OC biomarkers that help in predicting prognosis and guiding effective targeting therapy are improved.

Tumor cells feature a switch in metabolism from mitochondria-based aerobic oxidation to cytoplasm-based glycolysis even under normoxia conditions, referred to as the “Warburg Effect” 6, which supplies efficiently biosynthesis materials such as NADPH and glutathione for cancer cells 7. Therefore, Warburg Effect becomes one of the hallmarks of cancer, and targeting cell metabolism disruption specifically could be a promising strategy in cancer therapy 8.

Glucose metabolism is a process of transmitting from cytosol to mitochondria and mitochondria are pivotal in deciding the destiny of a cell since pyruvate participating in oxidative decarboxylation of mitochondria to produce acetyl coenzyme A (acetyl-CoA) becomes the key point 9. PDC, as a gatekeeper of glucose oxidation, converts pyruvate to acetyl-CoA, which bridges glycolysis with the Krebs cycle 10 and is regulated by phosphorylation (pyruvate dehydrogenase kinases, PDKs) and dephosphorylation (pyruvate dehydrogenase phosphatases, PDPs) events. Recent studies have suggested that the cancer-specific metabolic key enzyme such as PDCs 11 and PDKs 12 could be designed as potential therapeutic targets in diversified anticancer discovery efforts.

PDK is a mitochondrial enzyme that inhibits PDC catalyzing the decarboxylation of pyruvate to acetyl-CoA by phosphorylation 13. Inhibition of PDK resulting in the activation of oxidative phosphorylation has turned out to be a feasible therapeutic strategy to reverse Warburg effect and restrain cancer cells proliferation 14. Four isoforms, PDK1, PDK2, PDK3, and PDK4, have been isolated and identified in human cells, among them, PDK1 is the unique isoform reported to phosphorylate all three serines (Ser232, Ser293, and Ser300) 15 and implicated in cancer malignancy 16. It had been reported that PDK1 was remarkably overexpressed in various tumors such as lung cancer 7, gastric cancer 17,glioblastoma 18, etc. 19-25, regulated by hypoxia-inducible factor-1α (HIF-1α) mediated 26 or Wnt/β-catenin signaling 27 and contributed to chemoresistance of ovarian cancer by activating EGFR 28. Michelle K. Y's team had confirmed that PDK1 could promote the development of ovarian cancer especially in metastasis by modulating tumor-mesothelial adhesion, invasion, and angiogenesis via α5β1 integrin and JNK/IL-8 signaling 29. However, its expression in ovarian cancer tissues compared with benign ovarian tumor, its correlation with clinicopathological features and prognosis, and its influence on biological behavior of ovarian cancer cells have not been unknown up to now.

In the present study, we profiled the expression status of PDK1 in the ovarian cancer and evaluated the prognostic significance of PDK1 in metastasis of ovarian cancer. We found PDK1 was an independent risk factor affecting metastasis of ovarian cancer, and could be useful in ovarian cancer risk assessment and future therapeutic targeting.

## Materials and methods

### Patients and Specimens

This research was approved by the Ethical Committee of the First Affiliated Hospital of Nanjing Medical University (Nanjing, China) (Ethics: 2017-SRFA-064). All patients were informed experiment consent which included they have the right to withdraw from the experiment in the course of the experiment. The criterion of our selection of specimens is those who were staged according to the International Federation of Gynecology and Obstetrics by pathologists and excluded cases who received an operation, radiotherapy, or preoperative chemotherapy before collecting informed consent. Fresh tumor tissues were collected from OC patients (n=17) and benign ovarian tumor (BOT) patients (n=15) treated at the First Affiliated Hospital of Nanjing Medical University. For immunohistochemistry assays, OC tissues (n=88) and BOT (n=46) samples were obtained from the pathology department of Nanjing Maternity and Child Health Care Hospital, Nanjing Drum Tower Hospital, Jiangsu Cancer Hospital and Nanjing Jiangning hospital from January 2011 to August 2017. All tissue sections were diagnosed by pathologists. The clinical data of the patients was also collected for analysis. Patient's demographic and clinicopathological features are shown in Table 1.

### Immunohistochemistry and Scoring

PDK1 expression levels between OCs and BOTs were semi-quantitatively compared by immunohistochemistry (IHC) scoring. Briefly, slides were dried in the oven at 70 °C for 40 min and dehydrated in xylene and graded alcohols. Antigen retrieval was performed with 0.01 M citrate buffer at pH 6.0 at 95 °C for 20 min. After cooling to room temperature, the slides were incubated with 3% hydrogen peroxide to block endogenous peroxidase activity. Then slides were incubated with PDK1 antibody (1:100, ab110025, Abcam Inc, Cambridge, MA, USA) at 4 °C overnight followed incubating with horseradish peroxidase-linked secondary antibodies at 37 °C for 15 min and stained using diaminobenzidine solution and counterstained with hematoxylin. Two independent pathologists with no prior knowledge of clinicopathologic information evaluated the IHC results.

IHC values for PDK1 were calculated by multiplying immunostaining intensity by the proportion of positive cells. The intensity was scored on a 4-point scale for cytoplasmic staining: 0 = no reactivity; 1+ = faint or weak reactivity; 2+ = moderate reactivity and 3+ = strong reactivity. The proportion of positive cells over the total number of cells was semiquantitatively evaluated and classified into 5 scores, as follows: score of 0: 0%, score of 1: 1-25%, score of 2: 26-50%, score of 3: 51-75%, and score of 4: >75%. The final score was obtained by multiplying the intensity score to proportional scores and was classified as follows: 0-2 as negative and 3-12 as positive.

### Cell Lines and Cell Culture

Human ovarian cancer cell lines, CAOV3 and SKOV3, were ordered from American Type Culture Collection (Manassas, VA, USA). The normal cell line used in this study was human ovarian surface epithelial cell line (HOSEpiC) obtained from ScienCell Research Laboratories (San Diego, CA). SKOV3 cells were grown in McCoy's 5A (Invitrogen, Carlsbad, CA) medium. Moreover, CAOV3 and HOSEpiC cells were grown in Dulbecco's Modified Eagles Medium. All above mediums were supplemented with 10% fetal bovine serum (FBS; Invitrogen, Carlsbad, CAUSA), 100 U/mL penicillin, and 100 μg/mL streptomycin and incubated in a humidified atmosphere at 37 °C with 5% CO_2_.Moreover, cells were cultured in antibiotic-free medium before transfection.

### miRNA Transfection

PDK1 small interfere RNAs (siRNA-PDK1) and negative control's (siRNA-vector) were purchased by Santa Cruz Inc and diluted to 10 μmol/L according to instruction before use. CAOV3 and SKOV3 cells were transiently transfected with 20 nmol/L of siRNA-PDK1/siRNA-vector by using lipofectamine^TM^ RNAiMAX Transfection Reagent (Invitrogen, Carlsbad, CA, USA) when grew to reach about 50%-60% confluence according to the manufacturer's protocols. Following 5.5 h transfection, the media was removed and the cells were placed in serum-containing medium and maintained at 37 °C in an atmosphere of 5% CO_2_. After 24 hours, the transfected cells were collected and processed for proliferation, invasion, cell apoptosis and cell cycle assay. After 48 hours, the transfected cells were collected and processed for wound healing and colony formation assay. Knockdown efficiency was examined by real time PCR and Western Blot after 48 h and 72 h transfection, respectively.

### RNA Isolation, Reverse Transcription and Real time PCR

Total RNA was isolated from the cultured cells, fresh OC and BOT tissues using the miRNeasy Mini Kit (Qiagen, Germantown, MD, USA) and quantified. cDNA was synthesized from 1 g/L RNA followed by PrimeScript RT Master Mix (Takara, Otsu, Japan) standard protocol. The expression levels of PDK1 were examined by performing real time PCR with an SYBR-Green in conjunction with an ABI 7500 system (Life Technologies, Foster City, CA, USA). The primer sequences used are summarized in Table 2. PCR conditions consisted of 30 seconds at 95.0 °C 1 cycle, 5 seconds at 95.0 °C, 30 seconds at 57.0 °C and 34 seconds at 72.0 °C 52 cycles, 5 min at 72.0 ℃ for holding stage. The Cycle threshold (CT) values were estimated by normalizing these values against β-action CT values using the 2^-ΔCt^ for comparison of tissues and 2^-ΔΔCt^method for comparison of cell lines.

### Western Blot

Total cells collected from different experiments were obtained by lysing in RIPA buffer. The protein concentration was determined using the BCA Protein assay kit (Pierce Biotechnology, Rockford, IL, USA). Cell lysates were subjected to SDS-PAGE electrophoresis and proteins were blotted onto PVDF membranes. The membranes were further incubated with primary antibodies PDK1 (1:1500, ab110025, Abcam Inc, Cambridge, MA, USA) and GAPDH (1:2000, AF0006, Beyotime Biotechnology, Shanghai, China) followed by incubation with an HRP‑conjugated secondary antibody (1:3000, A0216, Beyotime Biotechnology, Shanghai, China). Exposure was performed using enhanced chemiluminescence reagents (Millipore, Billerica, MA, USA).

### Cell Proliferation Assay

Cell proliferation detection was assessed by Cell Counting Kit-8 (CCK8) cell proliferation kit (Tongren, Shanghai, China) according to the manufacturer's instructions. Briefly, cells transfected with siRNA for 24 hours were plated into 96-well plates at 3×10^3^ cells/well with 100 μL complete medium and cultured under normal conditions. At the indicated time (24, 48 and 72 hours), cells were incubated with 100 μL of complete medium plus 10 μL of CCK8 reagent at 37 °C for 2 hrs. Then, the absorbance was measured on a microplate reader (Bio-Rad, La Jolla, CA, USA) at a wavelength of 450 nm.

### Cell Invasion Assay

Cell invasion assay was performed by a 24-well Transwell chamber with a pore size of 8 μm (Sigma, San Francisco, USA). Cell starvation was incubated for 8 hours before the experiment began. Each chamber was pretreated with 70 μL Matrigel (BD Biosciences, NJ, USA) diluted by the serum-free DMEM medium at the ratio of 1:10. The cells fixed with medium which containing 0.2% FBS were trypsinized after transfection for 24 hours and transferred to the upper Matrigel chamber in 100 μL of serum-free medium supplementing 5×10^5^ cells and incubated for 48 hours, the bottom chamber was filled with medium containing 30% FBS. The chambers were incubated for 48 h at 37 °C in 5% CO_2_ atmosphere. The non-migrated cells were removed by cotton swab, then fixed with 95% ethanol for 30 min and stained with 0.1% crystal violet at 37 °C for 25 min, washed twice with PBS. The cells migrated through the chamber were observed by microscope (×40 magnification and ×100 magnification).

### Wound healing Assay

For the wound healing assay, siRNA-PDK1 and siRNA-vector were transfected into the cells in 6-well plates. The cell layers were then scratched using a 200 µL sterile pipette tip to form wound gaps. The wound location in the 6-well plates was marked. Images of cells were captured to record the wound width at the marked wound locations at 0, 12 and 24 h in order to measure the migratory ability of the cells. Migration index = (A_0h_ - A_12h/24h_) / A_0h_. Scratch area (Area, A) was measured by Image Pro Plus software.

### Colony Formation Assay

Transfected CAOV3 and SKOV3 cells (3×10^3^) were seeded into Culture dish and cultured in cell culture medium for 14 days to allow colony formation. The culture medium was changed each 5 days. The colonies were then fixed in 100% methanol for 5 min and stained with 1.0% crystal violet solution for 15 min. The number of macroscopically detectable colonies was registered.

### Cell Apoptosis Assay

CAOV3 and SKOV3 cells were digested with trypsin without EDTA. The cells were harvested and diluted to a density of approximately 1×10^6^ cells/ml after 24 h transfection, then washed twice with ice-cold phosphate-buffered saline. Subsequently, the cells were incubated in the dark, at room temperature for 15 min with 5 µL phycoerythrin (PE) Annexin V, 5 µL 7-aminoactinomycin D (7-AAD) and 500 µL 1×binding buffer from the Annexin V-PE/7-AAD apoptosis kit (KeyGEN Bio TECH Corp, China) according to the manufacturer's protocol, then they were analyzed by flow cytometry. Cells undergoing early apoptosis only bound to PE Annexin V, whereas cells that bound to PE Annexin V and 7-AAD were in the late stages of, or had already undergone, apoptosis.

### Cell Cycle Assay

The CAOV3 and SKOV3 cells, treated with siRNA-PDK1 or siRNA-vector after 24 h, were collected (centrifugation at 1000 rpm for 5 min) and washed with PBS, then fixed in 70 % ethanol at 4 °C for 24 h. Then, the cells were centrifuged and washed with PBS twice. At last, cells incubated with 500 μL PI staining working fluid which included PI stain buffer, PI stain fluid and RNase A solution at 37 °C for 30 min. The samples were measured at the wavelength of 488 nm by flow cytometry (Beijing Leagene Biotechnology, China).

### Statistical Analysis

All experiments were performed at least three times, and all samples were analyzed in triplicate. All data were analyzed using SPSS 20.0 software and results are presented as means ± SD. Qualitative data were expressed as number (No.) and percent (%). Unpaired or paired two-tailed Student *t*-test was used to compare two groups of normally distributed data. Mann-Whitney *U* test was used for non-normally distributed data. Two-way ANOVA was used to analyze the interaction between two factors.

Criteria for judging high and low expression were median in age, size, PDK1 level and CA125 level group. It should be noted that serum CA125 levels were measured before ovarian cancer surgery. Association between clinicopathological features and immunohistochemical variables was evaluated by chi-square or Fisher exact test. Survival curves were plotted using the Kaplan-Meier product limit method, and significant differences between survival curves were determined using the log-rank test. A Cox regression proportional hazards model was used for univariate and multivariate analyses to identify independent prognostic factors. *P* < 0.05 was considered statistically significant.

## Results

### The expression of PDK1 was the highest in ovarian cancer cell lines among the four isoenzymes

**Figure 1:**
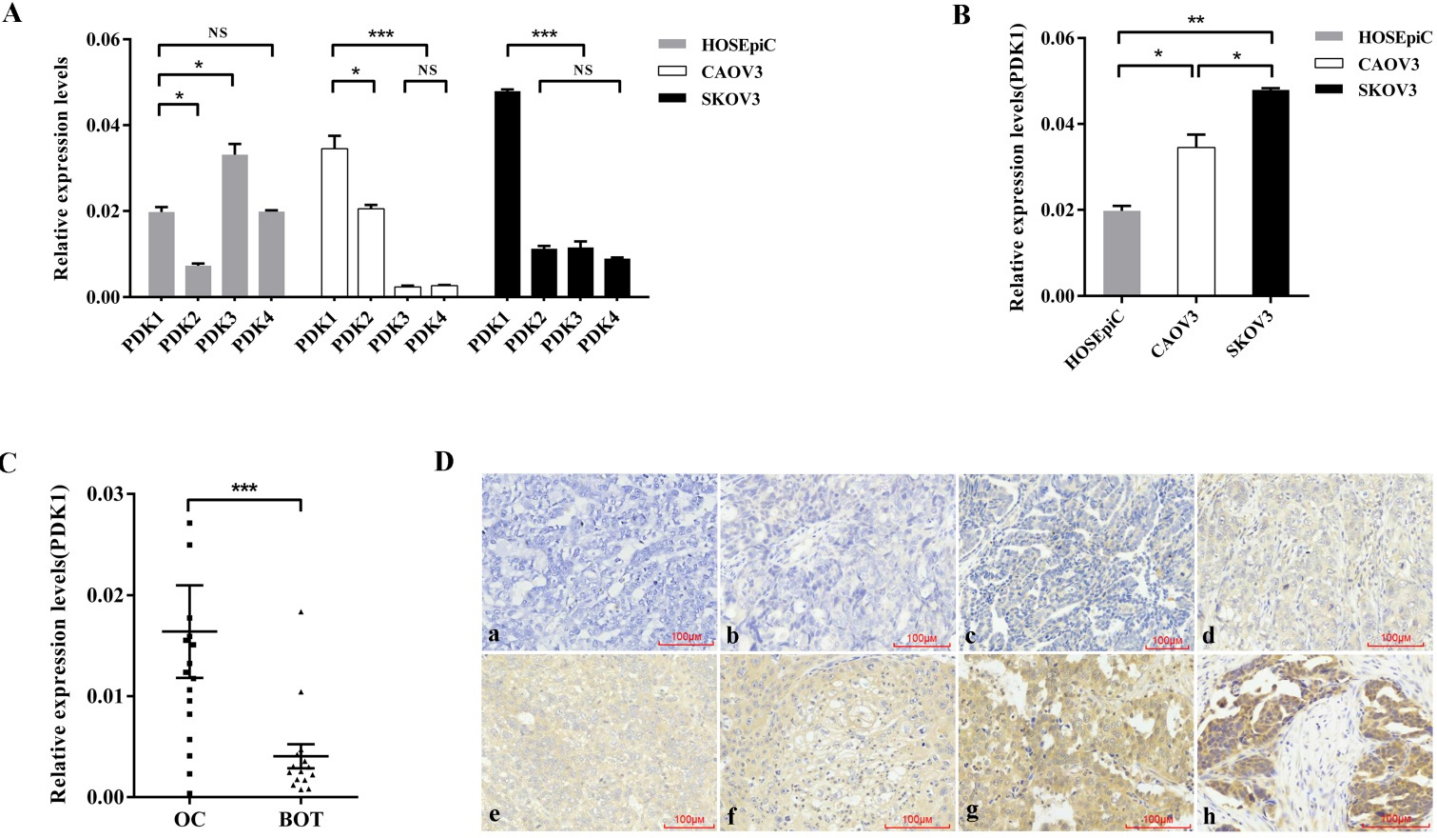
** PDK1 is overexpressed in ovarian cancer. (A)** Expression of four isoforms of PDK in ovarian cancer cell lines, CAOV3 and SKOV3, and normal ovarian epithelial cell line, HOSEpiC.** (B)** PDK1 was highly expressed in CAOV3 and SKOV3 cells compared with HOSEpiC cells.** (C)** PDK1 was highly expressed in ovarian cancer tissues compared to benign ovarian tumors.** (D)** Immunohistochemical detection of PDK1 protein expression in paraffin-embedded tissues. Positive PDK1 staining was observed mainly in the cytoplasm of OC tissues; a, IHC scoring = 0; b, IHC scoring = 2; c, IHC scoring = 3; d, IHC scoring = 4; e, IHC scoring = 6; f, IHC scoring = 8; g, IHC scoring = 9; h, IHC scoring = 12; a and b, PDK1 was not detected in OC tissues; c and d, representative images of weak PDK1 staining in OC tissues; e and f, representative images of moderate PDK1 staining in OC tissues; g and h, representative images of strong PDK1 staining in OC tissues; original magnification ×200.

In order to explore the expression of four PDK isoenzymes in ovarian cancer cell lines, CAOV3 and SKOV3 compared with normal ovarian epithelial cell lines, HOSEpiC, we used real time PCR to detect the RNA expression of *PDK1*, *PDK2*, *PDK3* and *PDK4*. The results showed that compared with *PDK2*, *PDK3* and *PDK4*, *PDK1* was aberrantly overexpressed in ovarian cancer cell lines, CAOV3 and SKOV3, while the expression of *PDK1* was higher than *PDK2*, but lower than *PDK3* and *PDK4* in normal ovarian epithelial cell line, HOSEpiC (Figure 1A). As shown in Figure 1B, *PDK1* expression was significantly higher in CAOV3 and SKOV3 cells, compared with HOSEpiC (*P* < 0.05), and the highest expression was found in SKOV3 cells.

### Highly expression of PDK1 closely associated with tumor size, FIGO stage, extraovarian metastases status and distribution

To assess the variant expression of the key enzyme in glucose metabolism, expression levels of *PDK1*were investigated in 17 ovarian cancer tissues compared with 15 benign ovarian tumors by real time PCR. The result as shown in Figure 1C was that PDK1 expression levels were significantly higher in ovarian carcinoma tissues in comparison with that in BOTs (*P* = 0.0008).

In order to further confirm the fact that PDK1 is highly expressed in cancer tissues, we detected the expression and cellular distribution of PDK1 in the paraffin sections of 88 ovarian cancers and 46 benign ovarian tumors through IHC staining. Staining results are shown in Figure 1D and varied in the intensity and percentage of positive tumor cells. From the diagrams, tumor cells exhibited cytoplasmic immune reaction of PDK1 (Figure 1Da-1Dh). According to the semi-quantitative score for both intensity and percentage of positive staining, the positive reaction would be increased accompanied by the scores increasing. Positive expression of PDK1 in ovarian tumors is described in Table 3. According to Table 3, the positive expression of PDK1 was more in malignant tumors (73/88, 83.0%) than benign (16/46, 34.8%), and this was statistically significant (*P* < 0.0001).

We assessed the correlation between PDK1 expression and clinical characteristics using expression levels obtained from IHC data of a cohort of 88 patients. We found that PDK1 expression level was significantly associated with tumor size (*P* = 0.002), FIGO stage (*P* = 0.024), extraovarian metastases status (*P* < 0.0001) and distribution (*P* = 0.006), while there was no significant correlation between age and differentiation and PDK1 expression (Table 4). Further multivariate logistic regression analysis revealed that tumor size (*P* = 0.002) and extraovarian metastases status (*P* < 0.0001) were independent risk factors for the expression of PDK1 in ovarian cancer (Table 5).

### PDK1 was a superior predictor of ovarian cancer prognosis and combining detection of PDK1 and CA125 can more effectively predict the prognosis of ovarian cancer patients

**Figure 2:**
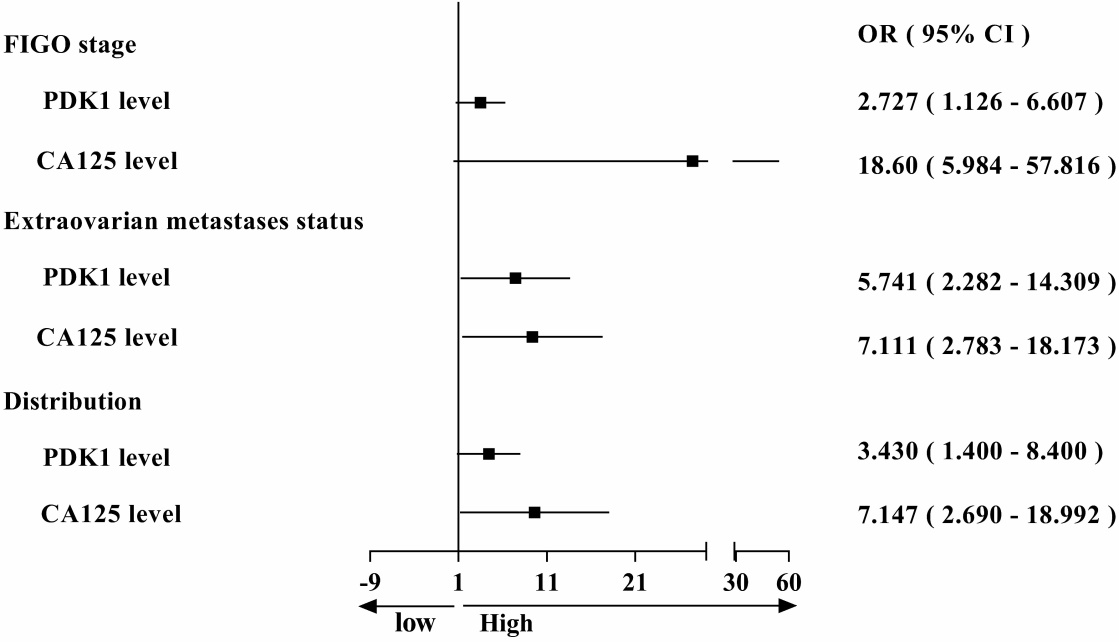
Comparison of odds ratio on FIGO staging, metastasis and distribution affected by PDK1 expression and CA125 level.

During the study, we also collected data on serum CA125 levels from 88 ovarian cancer patients at their first visit, and assessed the positive rate and correlation with clinical features in patients with ovarian cancer. We found that the positive rate of CA125 (84.09%) and the PDK1's (82.95%) in ovarian cancer patients was almost equal (Table 6). Then we also analyzed the correlation between serum CA125 level and clinicopathological features. Chi-square analysis revealed that the level of serum CA125 was closely related to differentiation (*P* = 0.013), FIGO stage (*P* < 0.0001), extraovarian metastases status (*P* < 0.0001) and distribution (*P* < 0.0001) of ovarian cancer (Table 4). To sum up, we found that both PDK1 and CA125 were closely related to FIGO stage, extraovarian metastases status and distribution, leading to comparative analysis of their risk factors as shown in Figure 2. From the figure, we concluded that the OR value is greater than 1, suggesting that both PDK1 and CA125 are risk factors for FIGO stage, extraovarian metastases status and distribution of ovarian cancer patients, and the OR value of CA125 is higher than that of PDK1, suggesting that the correlation between CA125 and FIGO stage (OR: 18.60 vs 2.727), extraovarian metastases status (OR: 7.111 vs 5.741) and distribution (OR: 7.147 vs 3.430) of ovarian cancer patients is stronger than that of PDK1. Further multivariate logistic regression analysis revealed that FIGO stage (*P* < 0.0001) and distribution (*P* = 0.029) were independent risk factors affecting the serum CA125 level in ovarian cancer (Table 5).

**Figure 3:**
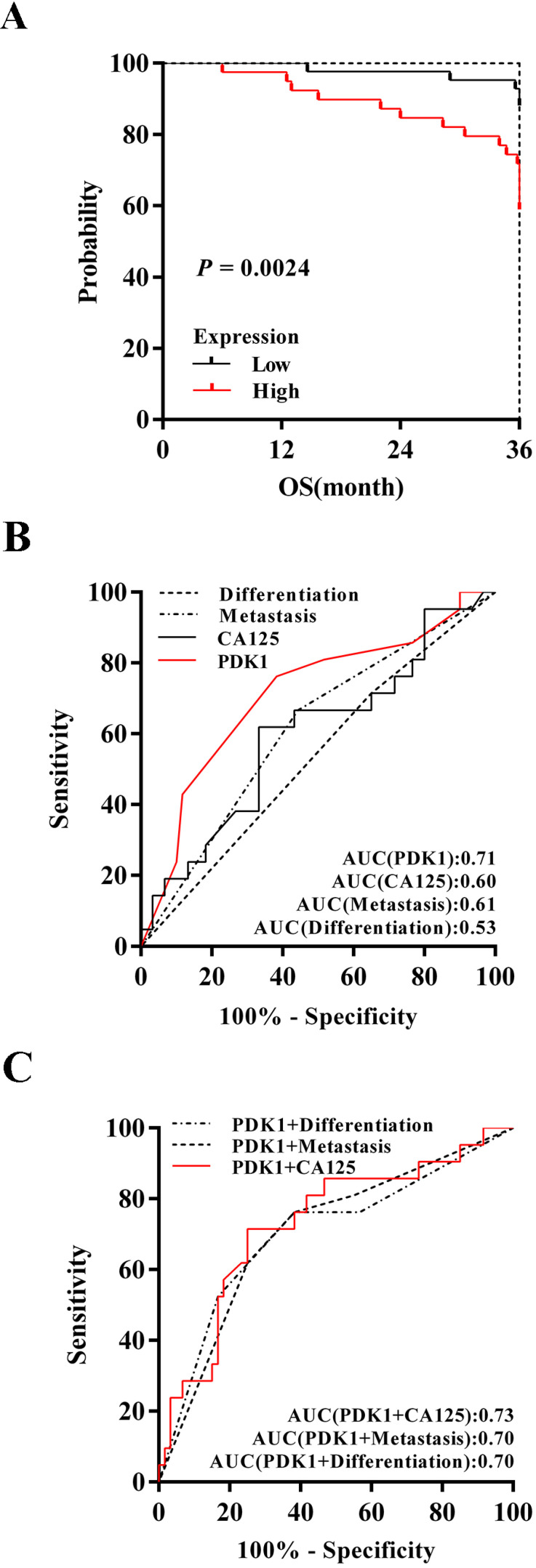
** PDK1 was a superior predictor of ovarian cancer prognosis and combining detection of PDK1 and CA125 can more effectively predict the prognosis of ovarian cancer patients. (A)** Kaplan-Meier (KM) survival curves comparing ovarian cancer patients stratified by expression of PDK1. **(B)** ROC analysis for evaluation of the accuracy of PDK1 protein level, serum CA125 level, metastasis status and tumor differentiation status for prognostic judgement of ovarian cancer.** (C)** ROC analysis for evaluation of the accuracy of PDK1 protein level combining serum CA125 level, metastasis status or tumor differentiation status respectively for prognostic judgement of ovarian cancer.

Kaplan-Meier survival analysis of 88 cases revealed a correlation between higher PDK1 expression levels and shorter overall survival time (*P* = 0.0024) (Figure 3A). Furthermore, inclusion of partial well-known clinicopathological factors with prognostic significance in our study would strengthen the utility of PDK1 as a prognostic predictor alone and in combination with other factors. The results of univariate survival analysis shown in Table 7. Factors that affected survival by univariate analyses included extraovarian metastases status and PDK1 (all *P* < 0.05). The patients' age, tumor size, tumor differentiation, FIGO stage, distribution, and serum CA125 level did not have a statistically significant effect on survival. In addition, in order to get a more precise combined analysis of all the factors and to control for confounding factors more effectively, all factors with *P* < 0.2 in univariate analyses were entered in a Cox proportional hazards model for multivariate survival analysis. When the effect of covariates was adjusted, the increase of PDK1 expression was an independent predictor of poor survival (HR = 4.088, 95 % CI: 1.496-11.165, *P* = 0.006) (Table 7). However, extraovarian metastases status, distribution and CA125 were affected by other factors that could not independently influence the probability of poor outcome.

To determine discrimination and superiority, the sensitivity and specificity of PDK1, extraovarian metastases status, distribution and CA125 were identified. The area under the receiver operating characteristic (ROC) curve was determined from the plot of sensitivity versus (1-specificity) [true positive rate versus false positive rate] and is a measure of the predictability of a test. A larger area under the ROC curve of 0.70 to 0.90 is considered superior discrimination, whereas a ROC value of 0.50 indicates no discrimination. ROC areas for PDK1, CA125, extraovarian metastases status and differentiation are 0.71, 0.60, 0.61 and 0.53 respectively (Figure 3B), which suggested that PDK1 has better discrimination ability. Furthermore, combining detection of PDK1 and CA125 can more effectively predict the prognosis of ovarian cancer patients as the optimal area under curve, 0.73 (Figure 3C).

### Down-regulation of PDK1 inhibited ovarian cancer cells proliferation, invasion, migration and colony formation

**Figure 4:**
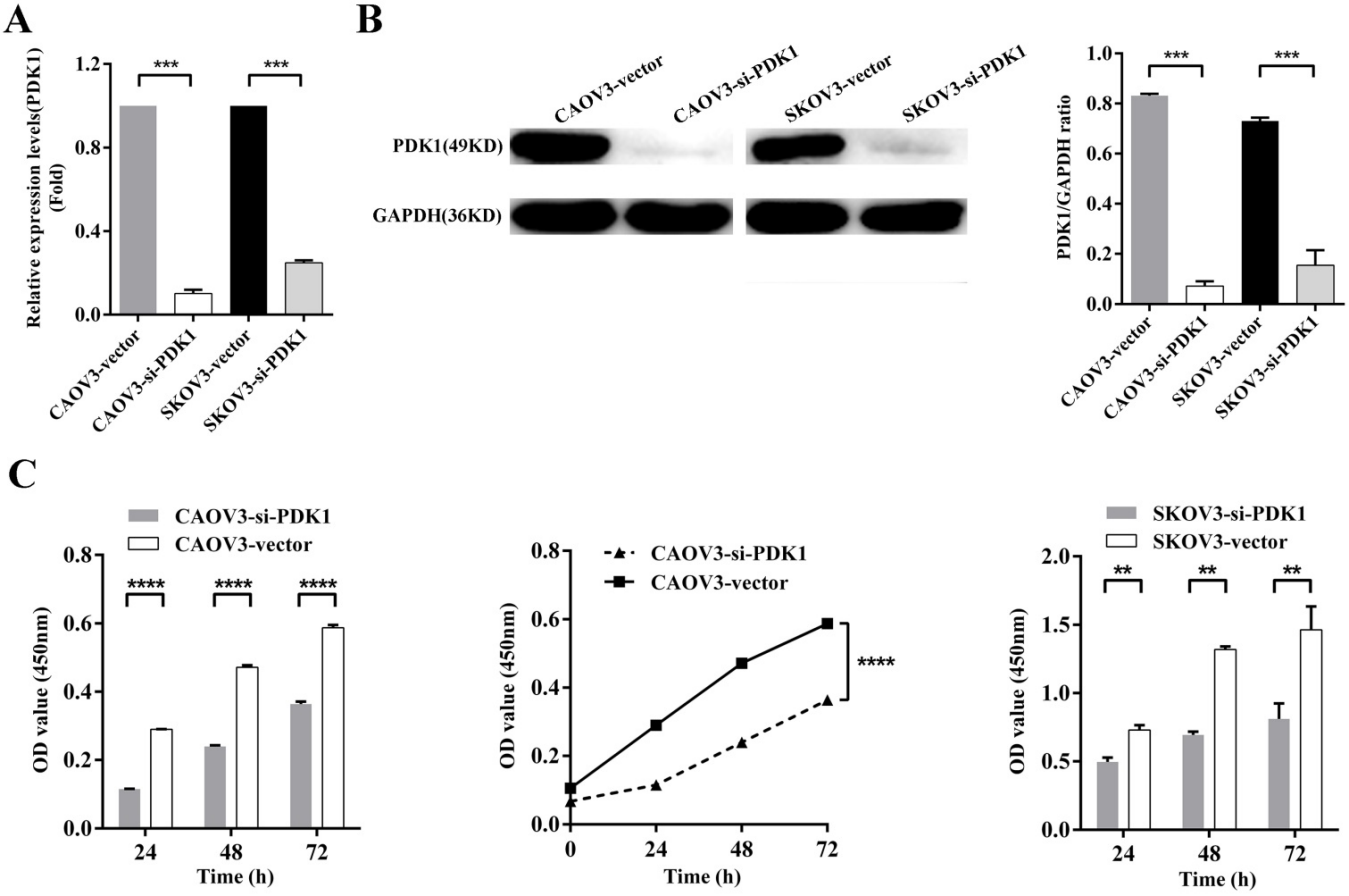

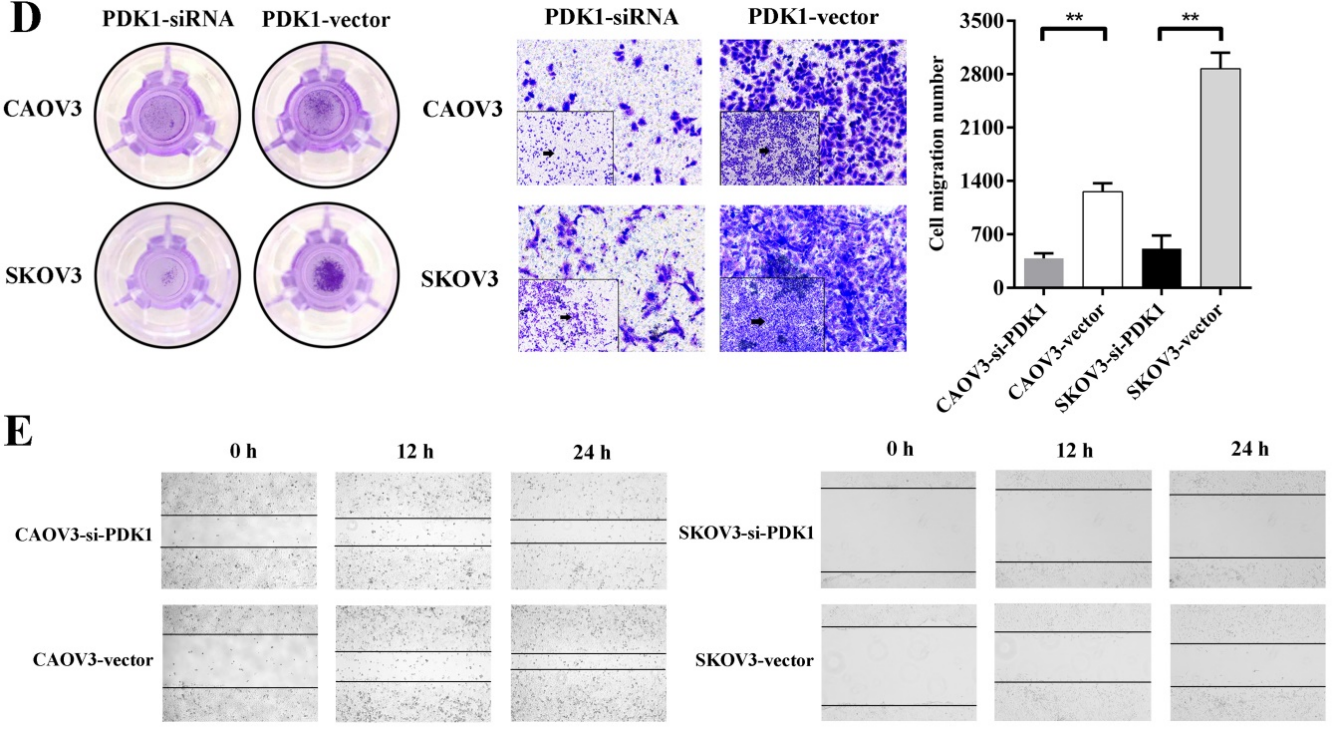

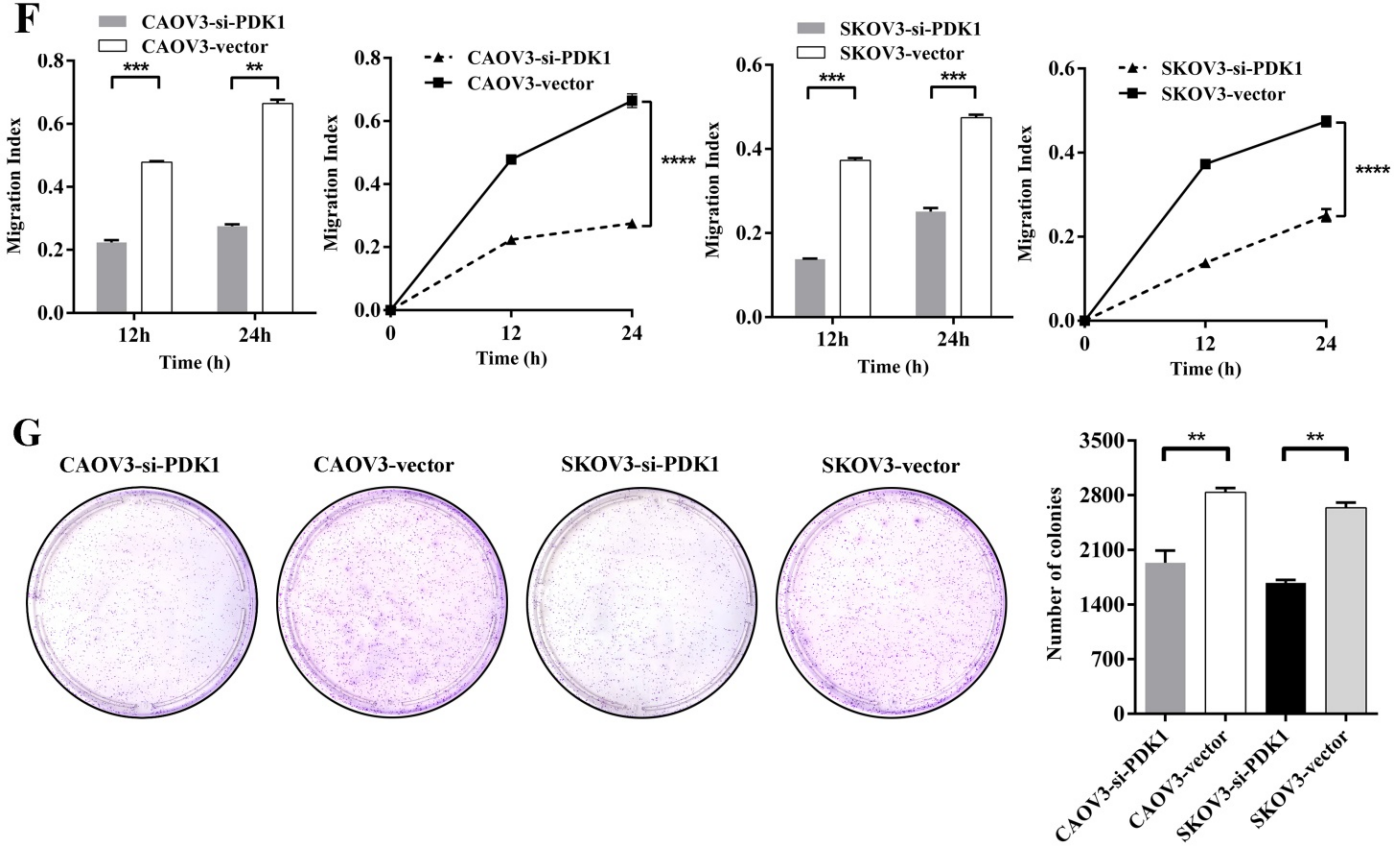
** Down-regulation of PDK1 inhibited ovarian cancer cells proliferation, invasion, migration and colony formation. (A)** CAOV3 and SKOV3 cells were treated with the siRNA-vector or siRNA-PDK1 for 48h. Then, PDK1 mRNA expression was analyzed by real time PCR.** (B)** Western blotting was used to further validate the interference effects. **(C)** The cells proliferation activity was detected by CCK8 assay. **(D)** The Transwell assay was used to detect cell invasion ability.** (E)** Cell migration was measured with wound healing assay after transfection for 12 h and 24 h. **(F)** Cell migration distances were calculated relative to the initial distance before migration and Two-way ANOVA was used to analyze the interaction between groups and time factors. **(G)** Colony formation assay was used to detect cell clonogenic ability.

To further examine whether PDK1 functions in ovarian cancer progression, *in vitro* studies were conducted. We knocked down PDK1 expression in CAOV3 and SKOV3 cells using small interfering RNAs; the most effective siRNA that showed more than 70% knockdown efficiency was selected for the following test. Figure 4A showed that mRNA expression after PDK1 depletion was decreased dramatically compared with the negative control's (*P* < 0.001). Western blot was used to further validate the knockdown efficiency and showed PDK1 level was declined notably after siRNA treatment in comparison with the negative control's (*P* < 0.001) (Figure 4B).

The effect of siRNA on cell proliferation was evaluated by CCK8 assay. The results indicated that the proliferation of silenced PDK1 cells was significantly decreased after 24h, 48h and 72h transfection compared with the negative control's cells (all *P* < 0.01). After analyzing the interaction between multiple factors, we found that there was interaction between group and time, and the difference was statistically significant which means PDK1 gene silencing aggravated the proliferation of CAOV3 and SKOV3 cells with the prolongation of siRNA reaction (*P* < 0.0001). Together, the above results suggested that siRNA-PDK1 markedly inhibits the proliferation of CAOV3 and SKOV3 cells (Figure 4C).

The ability of distant metastasis of tumor cells is the degradation of cell basement membrane by cell secreted proteolytic enzymes, which leads to cancer death and cell invasiveness is closely correlated with cancer metastasis. We therefore examined whether PDK1 knockdown affects invasiveness of ovarian cancer. A Matrigel invasion assay (Transwell invasion test) showed that PDK1 knockdown significantly inhibited invasiveness of CAOV3 and SKOV3 cells (Figure 4D). The number of migratory cells after PDK1 knockdown was significantly reduced in comparison with the negative control's group (*P* < 0.01).

To determine the effects of PDK1 on cell migration, wound healing assay was performed. Figure 4E indicated that CAOV3 and SKOV3 cells migration were dramatically decreased 24h after siRNA transfection. The relative migration index of cells transfected with siRNA group was sharply lower compared to the negative control's group (*P* < 0.0001). Through factors' interaction analysis, we found that interaction existed between groups and time, and the difference was statistically significant (*P* < 0.0001) (Figure 4F). These results indicate that siRNA-PDK1 considerably inhibits the migration of CAOV3 and SKOV3 cells and the inhibition of their migration became more obvious over time.

Just because PDK1 was obviously overexpressed in ovarian cancer, then we further investigated the effects of PDK1 on the ability of ovarian cancer cell growth and anchorage-independent growth. We demonstrated that the number of clones formed by siRNA-PDK1 treatment was significantly less than those by siRNA-vector treatment after 14 of day conventional culture (*P* < 0.01) (Figure 4G). It implied that PDK1 plays a critical role in the independent growth of ovarian cancer cells.

### Down-regulation of PDK1 arrested ovarian cancer cell cycle and induced ovarian cancer cell apoptosis

**Figure 5:**
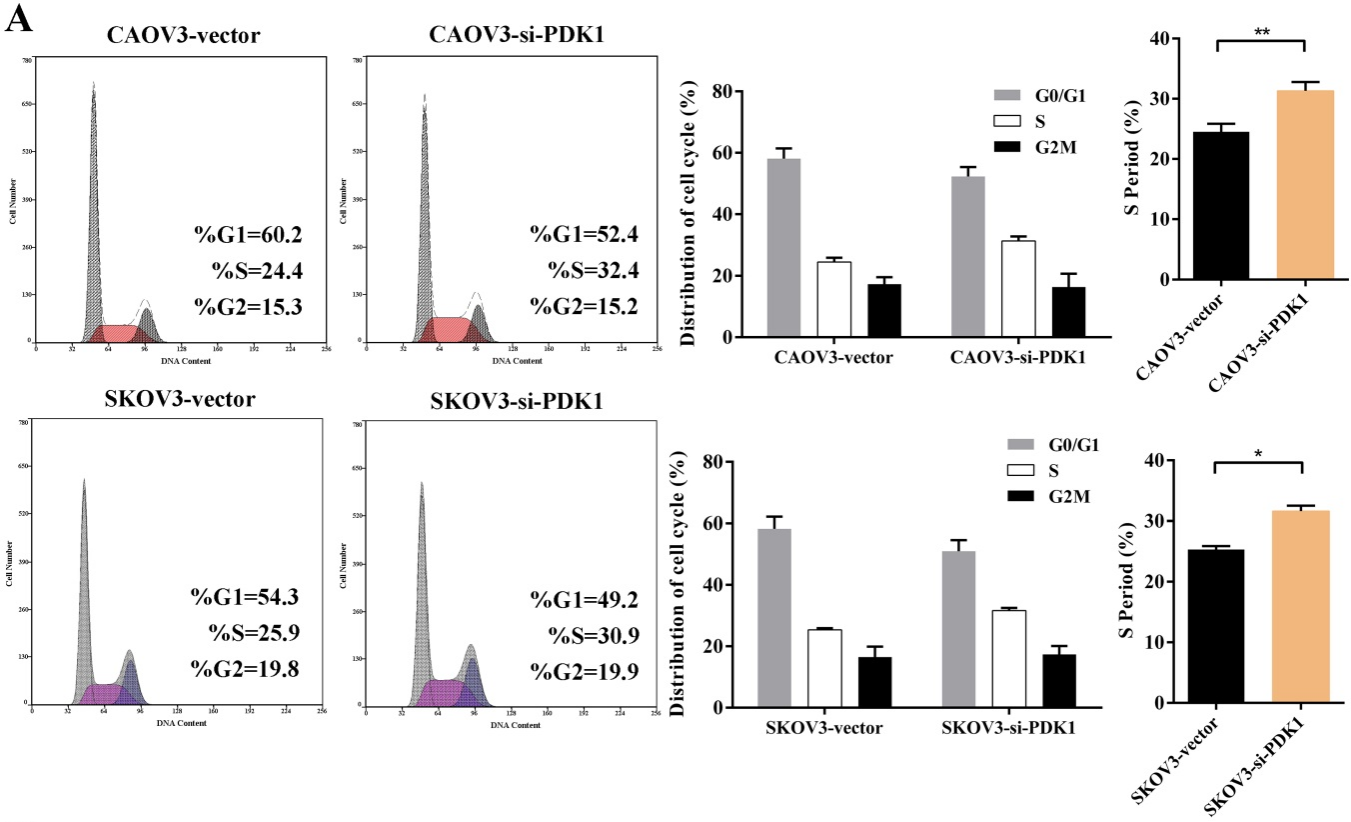

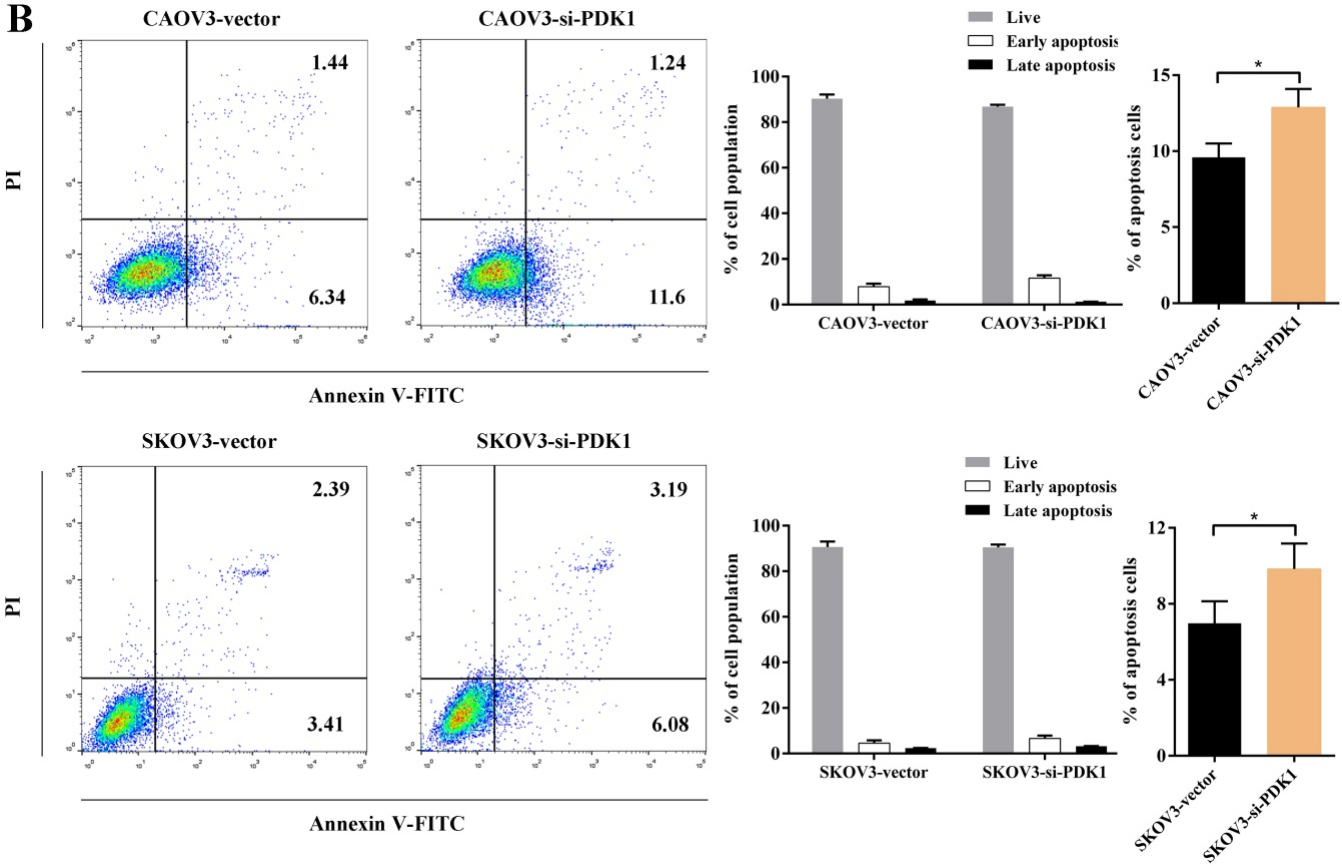
** Down-regulation of PDK1 arrested ovarian cancer cell cycle and induced ovarian cancer cell apoptosis. (A)** Cell cycle distribution of ovarian cancer cells analyzed by flow cytometry.** (B)** Annexin V/7-AAD staining was performed for apoptosis detection by flow cytometry.

Cell counting kit-8 (CCK8) and colony formation revealed down-regulation of PDK1 inhibited cell proliferation significantly compared with negative control. What is more, we investigated the effect of PDK1 on cell cycle. To be specific, we measured the percentage of tumor cells in different cell cycle stages including G_0_/G_1_, S and G_2_/M and evaluated the effect of PDK1 knockdown on the cell apoptosis in ovarian cancer cells by flow cytometry. As shown in Figure 5A, the results showed that in CAOV3 and SKOV3 cells transfected with siRNA-PDK1, the proportion of cells in the S phase was significantly higher than that in cells transfected with siRNA-vector (both *P* < 0.05), and there were no significant differences in the proportion of cells in the G_0_/G_1_ and G_2_/M phase between both groups. After siRNA-PDK1 was transfected into CAOV3 and SKOV3 cells, the cells exhibited significant cell cycle arrest. We further employed flow cytometry to analyze the effects of PDK1 on ovarian cancer cell apoptosis. The results showed that after siRNA-PDK1 transfection, the apoptosis rates in CAOV3 and SKOV3 cells were significantly increased compared with those in cells transfected with siRNA-vector (*P* < 0.05) (Figure 5B). This suggests that PDK1 knockdown induced S phase arrest in both CAOV3 and SKOV3 cells, thereby inducing cellular apoptosis.

## Discussion

Ovarian cancer is difficult to diagnose in early stage because of the special physiological location of the ovary, which leads to no obvious symptoms. Approximately 70% of cases are diagnosed at an advanced stage and extraovarian metastases have already occurred 30 which causes most of the death from advanced ovarian cancer. According to reports, 51% of ovarian serous cancers were diagnosed as stage III and 29% were diagnosed as stage IV from 2007 to 2013 in the United States, and their 5-year survival rates were 42% and 26%, respectively 5. Therefore, providing a perspective on novel promising approaches to biomarker discovery and their utility in ovarian cancer prognosis is very necessary and urgently needed.

In this study, we found that PDK1 was highly expressed in ovarian cancer cell lines, and the results were verified in ovarian cancer tissues. PDK1 is an inhibitor of key enzymes in glycose metabolism. Overexpression of PDK1 in cancer cells inhibits PDC-catalyzed tricarboxylic acid cycle, resulting in the inability of aerobic oxidation, may cause cancer cells prefer other material as their new energy source or other metabolic pathway to obtain large amounts of energy substances. Lee *et al.* 31 recently discovered in 2019 that the lymph node metastasis of tumor cells requires a metabolic shift toward fatty acid oxidation, suggesting a close relationship between metabolism and cancer metastasis, as well as a link between metabolism and prognosis, because lymph node metastasis predicts a poor prognosis of cancer patients. To further explore the factors affecting PDK1 expression, we compared the correlation between clinicopathological features and PDK1 expression, and concluded that tumor size and extraovarian metastases status are independent risk factors affecting PDK1 expression in ovarian cancer, which established the close relationship between PDK1-involved glycose metabolism and metastasis of ovarian cancer. According to Lee's research, we speculate that the high expression of PDK1 in ovarian cancer results in the inhibition of PDC in tumor cells, which drives the switch to fatty acid oxidation, promotes the metastasis of cancer cells and accelerates the adverse prognosis of ovarian cancer.

Since the serum CA125 level is well known as an indicator for clinical adjuvant diagnosis of ovarian cancer. Our results showed that the positive rates of PDK1 and CA125 in cases of ovarian cancer were almost the same. Previous studies have shown that serum CA-125 level can be considered as a clinical prognostic factor for survival and prognosis of patients with epithelial ovarian cancer after treatment, and it can help to detect pelvic or para-aortic lymph node metastasis 32, 33.In our study, serum CA125 level is closely related to differentiation, FIGO stage, extraovarian metastases status and distribution, and FIGO stage and distribution are independent risk factors for its high expression in ovarian cancer. Our unexpected finding that CA125 was not independently associated with metastasis is consistent with the latest findings 34. Because FIGO stage, extraovarian metastases status and distribution are closely related to the expression of PDK1 and CA125, we compared OR values, which are indicators of the intensity of association between exposure factors and disease. We found that CA125 overexpression group had higher correlation intensity than PDK1 in FIGO stage, extraovarian metastases status and distribution. These results suggest that when ovarian cancer patients develop metastasis, bilateral onset and advanced stage, the increase of CA125 is higher than that of PDK1.

Our research shows that PDK1 is the only biological target that can independently predict the prognosis of ovarian cancer among various factors affecting ovarian cancer. Although the correlation between CA125 level and disease characteristics is stronger than that of PDK1, PDK1 still exhibits excellent prognostic value, while CA125 does not, which also confirms the special role of metastasis in the prognosis of ovarian cancer. We observed that only metastasis and PDK1 were closely related to survival time and survival rate in Cox regression analysis. After multivariate analysis, PDK1 was the only biological target that could independently predict the prognosis of ovarian cancer patients, which was consistent with the results of previous influencing factors of PDK1.

Although current evidences show that various biological markers could predict the prognosis of ovarian cancer, such as CA125, HE4, CD24, CD44, CD133, SSEA and so on, few targets reported are related to tumor metabolism. Nowadays, the biological targets and inhibitors related to cancer metabolism, especially glycose metabolism, are of great clinical value not only in predicting the prognosis of cancer, but also in the treatment of cancer. Several studies have shown that metabolic signal targets of tumors provide promising means for new anti-cancer therapies 35-37.

Moreover, in this study, we developed a new predictive indicator system using integration of serum CA125 level into the PDK1 expression of ovarian cancer. Another interesting point found from this study is that the combination system improves the predictive value of OC survival over the single one.

Our study also showed that down-regulation of PDK1 inhibits tumor cellular proliferation, migration, invasion, growth and anchorage-independent growth ability, and the difference was statistically significant. From these results, we can deduce PDK1 may play a critical role in regulating biological behavior of ovarian cancer cells. Apoptosis and cell cycle experiments results revealed that PDK1 could affect the proliferation of tumor cells in S phase, in other words, replication phase. Currently, there are several latest research on PDK1 mechanisms involved in JNK/IL-8 signaling and PD-L1 pathway 29, 38, which have little correlation with tumor cell metastasis pathway. Together with previous studies, our current experiments demonstrated that PDK1 may participate in promoting the proliferation, invasion and metastasis of ovarian cancer cells. Although its molecular mechanism is still unclear, intervention can promote the cell cycle and apoptosis of cancer cells, involved by mitochondrial membrane potential (MMP) possibly 39. These findings will lead us to continue to explore the mechanism of PDK1 in ovarian cancer metastasis and strive to verify the expected results in biological sample bank, which will be our future goal and plan.

Above all, PDK1 was a superior prognostic biomarker of ovarian cancer and blocking PDK1 in adjuvant treatment of ovarian cancer is a promising antineoplastic therapy as other types of tumors 17, 23, 40, 41.

## Figures and Tables

**Table 1:** Clinicopathologic variables of 88 cases of ovarian cancer

Characteristics	Number (Proportion)
**Age (year)**	
Mean±SD	50.03±10.85
Median (Range)	50 (19-69)
<50 years	41 (53.4%)
≥50 years	47 (46.6%)
**Size (cm)**	
Mean±SD	7.31±4.42
Median (Range)	7.15 (1-19)
<7 cm	40 (45.5%)
≥7 cm	48 (54.5%)
**FIGO stage**	
I-II	36 (40.9%)
III-IV	52 (59.1%)
**Differentiation**	
Well	29 (33.0%)
Medium to poor	59 (67.0%)
**Extraovarian metastases status**	
No	44 (50.0%)
Yes	44 (50.0%)
**Distribution**	
Unilateral	53 (60.2%)
Bilateral	35 (39.8%)
**CA125 level in serum (U/mL)**	
Mean±SD	765.18±1517.91
Median (Range)	230.4 (2.17-10987)
< 230 U/mL	44 (45.5%)
≥ 230 U/mL	44 (54.5%)
**PDK1 level in tissue (Scores)**	
Mean±SD	6.14±3.56
Median (Range)	6 (0-12)
< 6 scores	46 (52.3%)
≥ 6 scores	42 (47.7%)

PDK1: Pyruvate Dehydrogenase Kinase 1; FIGO: The International Federation of Gynecology and Obstetrics; CA125: Carbohydrate Antigen 125.

**Table 2:** Primer sequence of the studied genes

Gene	Forward	Reverse
*PDK1*	CTGTGATACGGATCAGAAACCG	TCCACCAAACAATAAAGAGTGCT
*PDK2*	ATGAAAGAGATCAACCTGCTTCC	GGCTCTGGACATACCAGCTC
*PDK3*	CGCTCTCCATCAAACAATTCCT	CCACTGAAGGGCGGTTAAGTA
*PDK4*	GACCCAGTCACCAATCAAAATCT	GGTTCATCAGCATCCGAGTAGA
*β-actin*	GAGCTACGAGCTGCCTGACG	GTAGTTTCGTGGATGCCACAG

PDK1: Pyruvate Dehydrogenase Kinase 1; PDK2: Pyruvate Dehydrogenase Kinase 2; PDK3: Pyruvate Dehydrogenase Kinase 3; PDK4: Pyruvate Dehydrogenase Kinase 4.

**Table 3:** Expression of PDK1 in ovarian cancer and benign ovarian tumor

Tumor type	Total	PDK1 (semi-quantitative scores)				Positive Rate (%)	χ^2^	*P* value
		0-2	3-4	6-8	9-12			
ovarian cancer	88	15	21	35	17	83.0		
benign ovarian tumor	46	30	9	6	1	34.8	31.429	<0.0001

PDK1: Pyruvate Dehydrogenase Kinase 1.

**Table 4:** Correlations between the PDK1 expression in tumor tissues and serum CA125 level and the clinicopathologic features of OC

Clinical Variables	PDK1 expression in tissue		χ^2^	*P* value	CA125 level in serum		χ^2^	*P* value
	Low (%)	High (%)			Low (%)	High (%)		
**Age (year)**								
<50 years	26 (55.3)	21 (44.7)	0.375	0.540	22 (55.3)	25 (44.7)	0.411	0.521
≥50 years	20 (48.7)	21 (51.2)			22 (48.7)	19 (51.2)		
**Size (cm)**								
<7 cm	28 (70.0)	12 (30.0)	9.237	0.002	19 (70.0)	21 (30.0)	0.183	0.669
≥7 cm	18 (37.5)	30 (62.5)			25 (37.5)	23 (62.5)		
**Differentiation**								
Well	18 (62.1)	11 (37.9)	1.664	0.197	20 (62.1)	9 (37.9)	6.223	0.013
Medium to poor	28 (47.5)	31 (52.5)			24 (47.5)	35 (52.5)		
**FIGO stage**								
I-II	24 (66.7)	12 (33.3)	5.059	0.024	31 (66.7)	5 (33.3)	31.778	<0.0001
III-IV	22 (42.3)	30 (57.7)			13 (42.3)	39 (57.7)		
**Extraovarian metastases status**								
No	32 (72.7)	12 (27.3)	14.758	<0.0001	32 (72.7)	12 (27.3)	18.182	<0.0001
Yes	14 (31.8)	30 (68.2)			12 (31.8)	32 (68.2)		
**Distribution**								
Unilateral	34 (64.2)	19 (35.8)	7.536	0.006	36 (64.2)	17 (35.8)	17.126	<0.0001
Bilateral	12 (34.3)	23 (65.7)			8 (34.3)	27 (65.7)		

PDK1: Pyruvate Dehydrogenase Kinase 1; FIGO: The International Federation of Gynecology and Obstetrics; CA125: Carbohydrate Antigen 125.

**Table 5:** Multivariate logistic regression analysis of factors related to PDK1 expression and serum CA125 level of OC patients

The factors	PDK1 expression in tissue							CA125 level in serum						
	β	SE (b)	Wald *χ^2^*	*P* value	ORs	95%CI		β	SE (b)	Wald *χ^2^*	*P* value	ORs	95%CI	
						Lower	Upper						Lower	Upper
Size	1.715	0.541	10.041	0.002	5.555	1.923	16.042							
**Differentiation**								-0.188	0.707	0.071	0.791	0.829	0.207	3.316
FIGO stage	-0.461	0.695	0.441	0.507	0.631	0.162	2.460	2.541	0.601	17.877	<0.0001	12.697	3.909	41.240
Extraovarian metastases status	2.045	0.537	14.511	<0.0001	7.731	2.699	22.145	0.153	0.683	0.050	0.822	1.166	0.306	4.444
Distribution	0.707	0.606	1.360	0.243	2.208	0.618	6.650	1.257	0.577	4.744	0.029	3.514	1.134	10.889

PDK1: Pyruvate Dehydrogenase Kinase 1; FIGO: The International Federation of Gynecology and Obstetrics; CA125: Carbohydrate Antigen 125.

**Table 6:** The Positive Rate of PDK1 and CA125 in Ovarian Cancer

Name	Total	Negative	Positive	Positive Rate (%)	χ^2^	*P* value
PDK1	88	15	73	82.95		
CA125	88	14	74	84.09	0.003	0.96

PDK1: Pyruvate Dehydrogenase Kinase 1; CA125: Carbohydrate Antigen 125.

**Table 7:** Univariate/multivariate Cox proportional regression survival analysis

Variables	Univariate analysis			Multivariate analysis		
	HR	95% CI	*P* value	HR	95% CI	*P* value
Age (≥50 years vs <50 years )	1.344	0.497-3.640	0.560			
Size (≥6 cm vs <6 cm)	0.900	0.332-2.437	0.836			
Differentiation (Medium to poor vs Well)	1.346	0.455-3.985	0.591			
FIGO stage (III/IV vs I/II)	1.750	0.619-4.948	0.291			
Extraovarian metastases status(Yes vs No)	2.800	0.987-7.941	0.053	1.503	0.449-5.031	0.508
Distribution (Bilateral vs Unilateral)	2.043	0.746-5.594	0.165	1.316	0.478-3.621	0.595
CA125 (≥230 U/mL vs <230 U/mL)	2.125	0.768-5.882	0.147	1.732	0.715-4.194	0.224
PDK1 (High vs Low)	5.148	1.661-15.952	0.005	4.088	1.496-11.165	0.006

PDK1: Pyruvate Dehydrogenase Kinase 1; FIGO: The International Federation of Gynecology and Obstetrics; CA125: Carbohydrate Antigen 125.

## Abbreviations

OC: Ovarian Cancer
PDK1: Pyruvate Dehydrogenase Kinase 1
FIGO: The International Federation of Gynecology and Obstetrics
CA125: Carbohydrate Antigen 125
PDK2: Pyruvate Dehydrogenase Kinase 2
PDK3: Pyruvate Dehydrogenase Kinase 3
PDK4: Pyruvate Dehydrogenase Kinase 4
